# Florid plasmacytosis in a case of acute myeloid leukemia: A diagnostic dilemma

**DOI:** 10.4103/0971-5851.68853

**Published:** 2010

**Authors:** Aruna Rangan, Bhavna Arora, Pooja Rangan, Tina Dadu

**Affiliations:** *Department of Hematology, Sir Ganga Ram Hospital, Rajinder Nagar, New Delhi, India*; 1 *Department of Hematology, Kasturba Medical College, Manipal Academy of Higher Education, Mangalore, India*

**Keywords:** *Acute myeloid leukemia*, *myeloma*, *plasmacytosis*

## Abstract

The association of acute myeloid leukemia (AML) with plasmacytosis is a known, although rare event. There are very few case reports documenting an increase in the number of plasma cells at the time of AML diagnosis. Here, we present the case of a 65-year-old male diagnosed as acute myelomonocytic leukemia with exuberant plasmacytosis, which posed a difficulty in diagnosis. Paracrine interleukin-6 production by leukemic blast cells is thought to contribute to this associated reactive plasma cell proliferation.

## INTRODUCTION

Plasmacytosis is known in acute myeloid leukemia (AML) bone marrows undergoing remission after chemotherapy. However, cases reporting plasmacytosis at the time of AML diagnosis are relatively rare. Our patient presented with an exuberant proliferation of plasma cells on bone marrow examination, although an evident increase in blast cells led us to a final diagnosis of acute myelomonocytic leukemia. This case was a diagnostic dilemma on account of the confounding plasmacytosis.

## CASE REPORT

This 65-year-old man presented to our hospital with complaints of fever, occasional epistaxis and easy fatiguability for the past 5–6 months. Physical examination revealed no significant abnormality apart from pallor.

Blood counts were ordered, which showed pancytopenia with a hemoglobin of 10.0 g/dl, Total leukocyte count of 2,100/ul and platelets of 11,000/ul. Rouleaux formation was seen on smear. The erythrocyte sedimentation rate was 200 mmHg at the end of the first hour. X-ray chest showed infiltrative shadows. No abnormality was detected on ultrasound of the abdomen.

**Figure 1:**
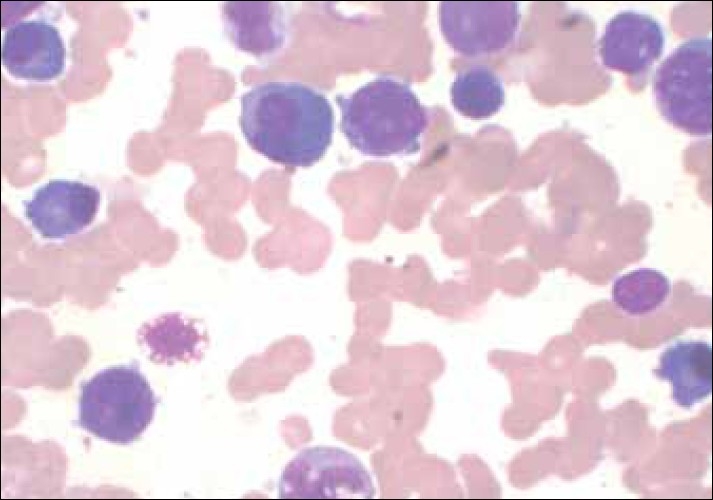
Bone marrow aspirate: Blast cells are seen along with many plasma cells (acute myelomonocytic leukemia) (Giemsa stain, 100×)

The bone marrow aspirate showed 22% blasts, with 25% mature plasma cells. Along with these cells were promonocytes 08%, myelocytes 06%, metamyelocytes 03%, polymorphs 02%, lymphocytes 08%, monocytes 10%, eosinophils 06% and nucleated red blood cells 10%. [Figure 1] The differential diagnosis of plamablastic myeloma and AML with reactive plasmacytosis were considered and further tests were performed to confirm the diagnosis.

**Figure 2:**
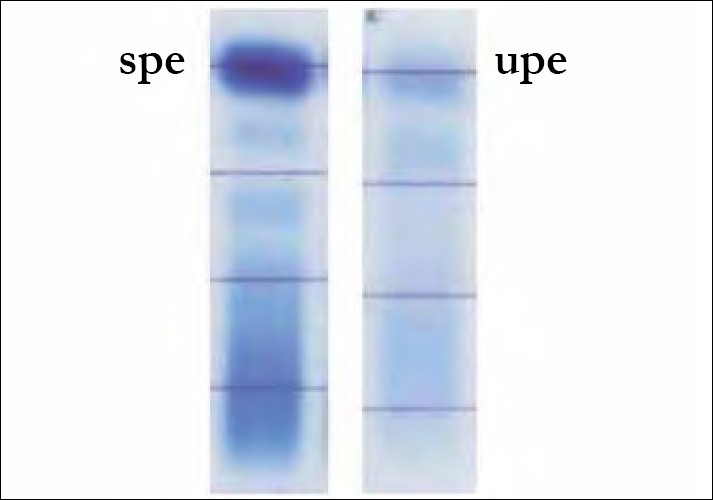
Serum and urine protein electrophoresis: Polyclonal rise in gamma globulins and nonselective proteinuria, respectively

Monoclonality was ruled out as the serum protein electrophoresis (SPE) showed a polyclonal expansion of gamma globulins. No monoclonal protein was found even on urine protein electrophoresis [Figure 2].

**Figure 3:**
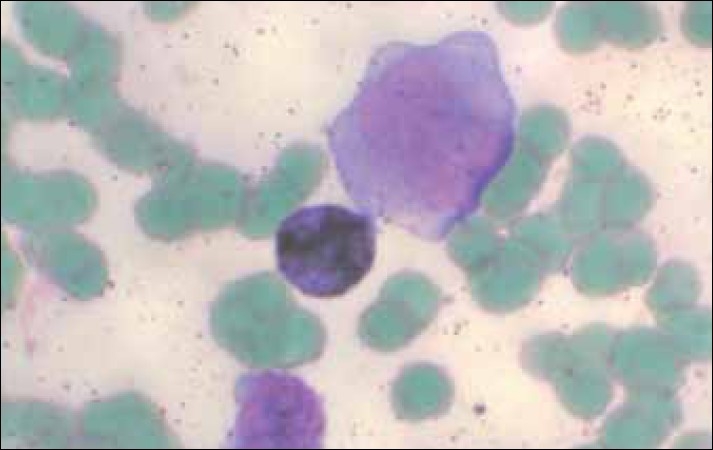
Myeloperoxidase stain: Myeloid blasts are positive (100×)

**Figure 4:**
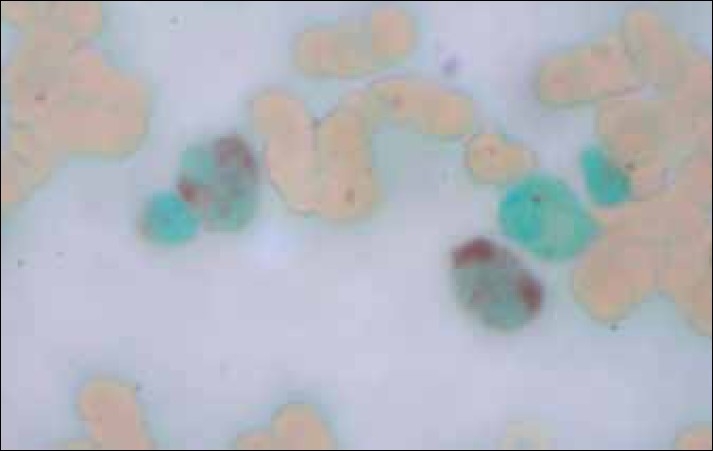
Alpha naphtyl butyrate esterase stain: Monocytic lineage is positive (monoblasts, promonocytes and monocytes) (100×)

Cytochemistry was performed to shed light on the nature of the blasts. Myeloperoxidase (MPO) stain [Figure 3] showed positivity in >3% of the blasts and alpha-naphthyl butyrate esterase (ANBE) was positive in most of the blasts thus identifying them as monocytic in origin [Figure 4].

Based on positive MPO and ANBE, and a polyclonal expansion of gamma globulins on SPE, a diagnosis of acute myelomonocytic leukemia (FAB subtype AML-M4) with reactive plasmacytosis was made.

## DISCUSSION

Reactive plasmacytosis characterized by a diffuse distribution of mature plasma cells in the bone marrow is known to occur in inflammatory conditions (bacterial and viral infections, collagen vascular diseases, granulomatous diseases, rheumatic heart disease), in liver cirrhosis and as a paraneoplastic syndrome in various neoplasms such as Hodgkin’s disease, non-Hodgkins lymphomas, carcinomas,[1–3] and in AML patients undergoing induction chemotherapy.[4]

The bone marrow of the 65-year-old man in our case showed a picture resembling a plasmablastic myeloma, with 25% plasma cells and 22% blast cells. It was only after some special stains (MPO, ANBE) and electrophoretic tests (including SPE and IFE) that a final diagnosis of AML-M4 with reactive plasmacytosis was arrived at.

Cases of AML that have a high plasma cell count can present as diagnostic dilemmas and should always be subjected to further sophisticated investigations before a diagnosis is given based only on morphology. Morphological features suggesting a reactive nature of plasma cells, although not specific, are mature forms of plasma cells, perivascular location of plasma cells and plasmacytic satellitosis (orientation of plasma cells around histiocytes).[5]

Few cases have been reported in the literature where plasmacytosis is seen with AML at the time of diagnosis.[5–7] In these cases, plasma cells usually do not exceed 10%. However, there are very few cases where the plasma cell count is found to be higher than 20% in newly diagnosed acute leukemias. In a study by Rosenthal *et al*.,[7] 149 cases of AML were studied at the time of diagnosis, with only two cases showing a plasma cell count of more than 20%.

The etiology of reactive bone marrow plasmacytosis is supposed to be a physiological response to antigenic stimulation.[1,2] Although mediation by a paracrine growth factor originating from the leukemic cells is possible, the mechanism of this bone marrow plasmacytosis has not yet been addressed. Interleukin (IL)-6 production by the AML blasts may play a pivotal role in the growth of the plasma cells.[5] For a definitive estimation of the role of IL-6 in AML-associated plasmacytosis, a larger cohort of patients should be analyzed.
